# Mirror-image Artifact of a Cephalhematoma Mimicking an Epidural Hematoma in a Newborn

**DOI:** 10.5334/jbsr.2783

**Published:** 2022-04-28

**Authors:** Elyn Van Snick, Bjorn Valgaeren, Bart Claikens

**Affiliations:** 1 Department of Radiology, General Hospital Damiaan, Ostend, Belgium

**Keywords:** mirror-image artifact, ultrasound, cephalhematoma, epidural hematoma, neonate

## Abstract

**Teaching Point:** When performing ultrasound examination of a cephalhematoma, the occurrence of a mirror-image artifact can mimic the presence of an epidural hematoma.

## Case History

A 3-day-old neonate was referred to the radiology department for ultrasonographic evaluation of a parieto-occipital cephalhematoma. The neonate was born through spontaneous vaginal delivery and no forceps nor suction cup was used. There were no clinical problems other than the presence of the cephalhematoma, and neurologic findings were normal.

**Figure 1:**
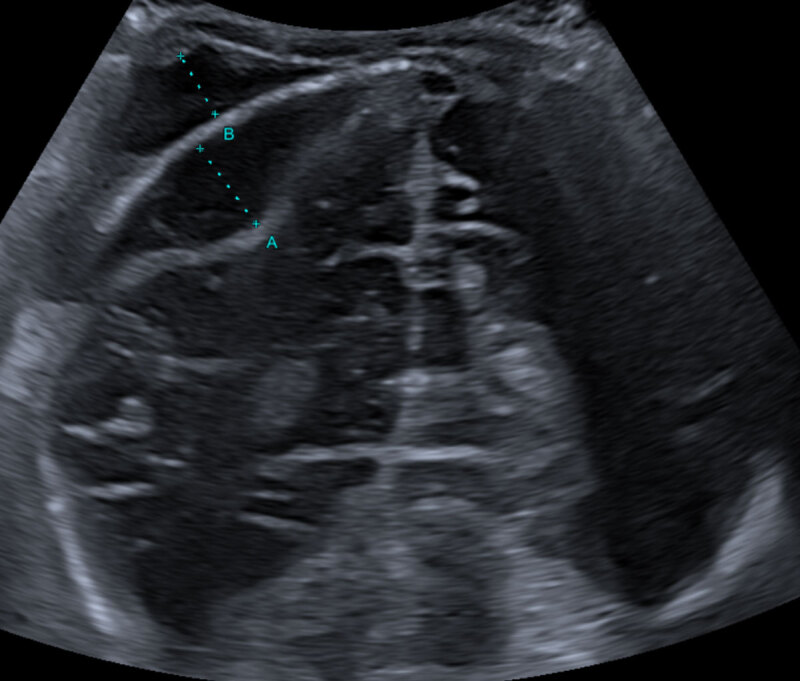


Coronal grayscale ultrasound image (***Figure 1***) showed the presence of an anechoic biconvex structure (dotted line A) resembling an epidural hematoma in the parietal region underlying the cephalhematoma (dotted line B). This was a curious finding because the delivery hadn’t been traumatic and there were no neurological symptoms present.

**Figure 2:**
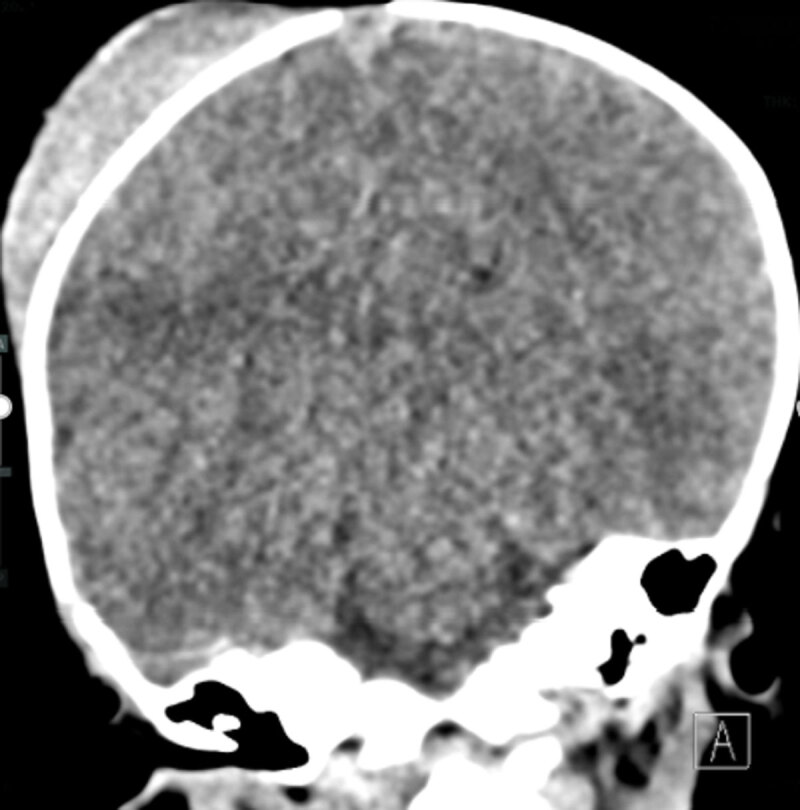


Because angulation of the probe could not clearly eliminate the possible presence of an epidural hematoma, in consultation with the treating paediatrician it was opted to further evaluate the infant through non-contrast-enhanced computed tomography (NECT) of the brain. Coronal NECT image (***Figure 2***) at the same level as the ultrasound image in ***Figure 1*** shows the cephalhematoma without underlying intracranial abnormalities.

The ultrasound findings were attributed to the occurrence of a mirror-image artifact.

## Comment

**Figure 3:**
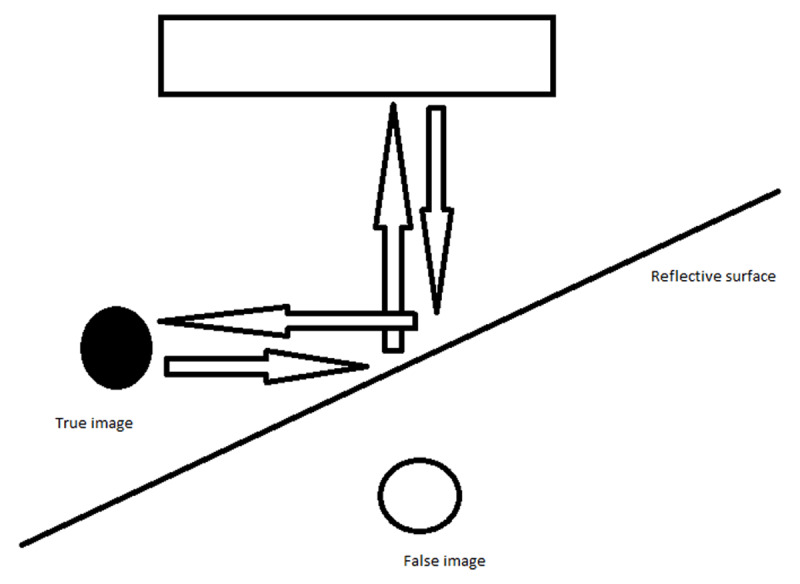


Mirror-image artifacts occur when the primary beam encounters an obliquely oriented highly reflective surface and is reflected by this surface but encounters another structure in its path before being reflected to the transducer. The transducer interprets the delayed echo as being reflected from a structure deeper down and this creates the mirror-image artifact on the other side of the reflective surface (***Figure 3***) [1].

In this case the ultrasound beam is reflected by the skull bone and a mirror-image artifact of the cephalhematoma is created on the opposite side, mimicking an epidural hematoma.

Other common examples of mirror-image artifacts include reflection of ascites across the diaphragm mimicking pleural effusion and reflection of the gestational sac mimicking heterotopic pregnancy.

The mirror-image artifact may closely resemble the original structure, but it may also appear weaker, distort the image of the original structure, or appear on images that do not simultaneously show the original structure. It is important to recognize such mirror-image artifacts and not mistake them for pathology so that unnecessary additional examinations and anxiety in patients can be avoided.

The occurrence of a mirror-image artifact can be evaluated by changing the angle of the primary beam or in case of a cephalhematoma applying graded compression, which shows simultaneous compression of the true image and the false mirror image. Doppler ultrasound may also be used, showing the presence of normal cerebral blood flow within the false mirror image [1].
